# Moving on from clinical animal-derived surfactants to peptide-based synthetic pulmonary surfactant

**DOI:** 10.1152/ajplung.00186.2024

**Published:** 2024-10-15

**Authors:** Frans J. Walther, Alan J. Waring

**Affiliations:** 1 Department of Pediatrics, David Geffen School of Medicine, University of California Los Angeles, Los Angeles, California, United States; 2 Department of Medicine, David Geffen School of Medicine, University of California Los Angeles, Los Angeles, California, United States; 3 Lundquist Institute for Biomedical Innovation at Harbor-UCLA Medical Center, Torrance, California, United States

**Keywords:** lung surfactant, surfactant proteins B and C, peptide mimics, nasal continuous positive airway pressure, aerosol delivery

## Abstract

Research on lung surfactant has exerted a great impact on newborn respiratory care and significantly improved survival and outcome of preterm infants with respiratory distress syndrome (RDS) due to surfactant deficiency because of lung immaturity. Current clinical, animal-derived, surfactants are among the most widely tested compounds in neonatology However, limited availability, high production costs, and ethical concerns about using animal-derived products constitute important limitations in their universal application. Synthetic lung surfactant offers a promising alternative to animal-derived surfactant by providing improved consistency, quality and purity, availability and scalability, ease of production and lower costs, acceptance, and safety for the treatment of neonatal RDS and other lung conditions. Third-generation synthetic surfactants built around surfactant protein B (SP-B) and C (SP-C) peptide mimics stand at the forefront of innovation in neonatal pulmonary medicine, while nasal continuous positive airway pressure (nCPAP) has become the standard non-invasive respiratory support for preterm infants. nCPAP can prevent the risk of chronic lung disease (bronchopulmonary dysplasia) and reduce lung injury by avoiding intubation and mechanical ventilation, is a relatively simple technique and can be initiated safely and effectively in the delivery room. Combining nCPAP with noninvasive, preferably aerosol, delivery of synthetic lung surfactant promises to improve respiratory outcomes for preterm infants, especially in low-and-middle income countries.

## Graphical Abstract

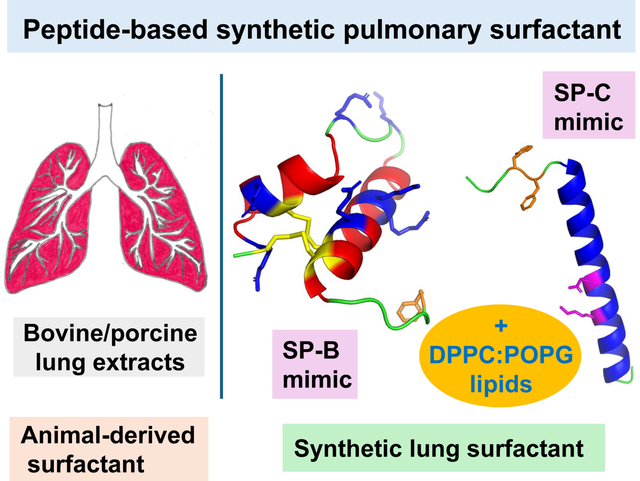

## INTRODUCTION

In 1957 John Clements (1) isolated saline-extractable surface-active material from animal lungs and two years later Avery and Mead (2) identified surfactant deficiency as the cause of respiratory distress syndrome (RDS) in preterm infants. We now know that mammalian surfactant is produced by alveolar type 2 cells and is a mixture of ~80–85% phospholipids, 10% neutral lipids and 8% protein that quickly lowers surface tension at the air-fluid interface in the alveoli, thereby keeping them open during expiration and stabilizing pulmonary gas exchange (3). The two most important phospholipids in surfactant are dipalmitoyl phosphatidylcholine (DPPC) and phosphatidylglycerol (PG). Organic solvent extracts of alveolar washes or lung tissue contain variable amounts (~1–2%) of the hydrophobic surfactant proteins (SP-B and C), but no hydrophilic surfactant proteins A and D (SP-A and D). SP-B and C are essential for surface activity and SP-A and D are part of native immunity in the lung. The presence of SP-B in surfactant is pivotal as SP-B deficiency is lethal (4). Processing and sterilization of these organic solvent extracts can affect structure and function of its components.

The first successful clinical trial with bovine surfactant treatment of 10 very preterm infants with RDS by Fujiwara in 1980 (5) led to the emergence of various bovine and porcine animal-derived surfactants. In combination with antenatal corticosteroid treatment and improved respiratory support techniques, clinical surfactant therapy significantly decreased mortality and air leak syndromes in preterm infants with RDS in the early 1990s (6). However, surfactant therapy did not reduce the incidence of chronic lung disease (bronchopulmonary dysplasia). This observation gave rise to a shift towards non-invasive respiratory support with nasal continuous positive airway pressure (nCPAP) (7) and avoidance of tracheal intubation at birth, if possible. In the wake of increased use of non-invasive respiratory support, endotracheal surfactant treatment is nowadays frequently administered during a short period of intubation followed by rapid extubation, known as the INSURE (INtubation-SURfactant-Extubation) method (8), or via a thin catheter, a technique described as less invasive surfactant administration (LISA) (9) or minimally invasive surfactant administration (MIST) (10). Laryngeal mask airway surfactant administration also avoids endotracheal intubation and ventilation (11), but still needs to find its niche in respiratory care of very preterm infants.

The composition of animal-derived surfactant formulations has not changed since their introduction four decennia ago. Current clinical surfactants include the bovine surfactants beractant (SURVANTA^®^, AbbVie Inc., North Chicago, IL 60064), calfactant (INFASURF^®^, ONY Biotech, Amherst, NY), neosurf (BLES^®^, Biochemicals, London, Ontario, Canada), and SF-RI 1 (ALVEOFACT^®^, Lyomark Pharma GmbH, Oberhaching, Bavaria, Germany), and the porcine surfactant poractant alfa (CUROSURF^®^, Chiesi Farmaceutici S.p.A., Parma, Italy). Early trials of synthetic surfactants without the vitally important surfactant protein B (SP-B) were disappointing. Progress in protein science and molecular modeling has enabled the development of advanced peptide mimics of SP-B and SP-C and synthetic surfactant formulations, i.e., CHF5633 (12), MINISURF^®^ (13), and SynthSurf (14), with surface activity that can compete with animal-derived surfactants. In parallel to the advances in surfactant design, novel delivery systems are being developed to enable effective aerosol delivery of synthetic surfactant during respiratory support with bubble nCPAP (15).

Considering the practical advantages of synthetic lung surfactants over their animal-derived counterparts (Table 1), we will focus on ongoing research on peptide-based synthetic lung surfactant and its advantages over currently available clinical, animal-derived surfactant formulations, including off-label use and their potential as a drug delivery system.

## SYNTHETIC LUNG SURFACTANT FOR PRETERM INFANTS WITH RDS

The first-generation of synthetic surfactants consisted mainly of lipids, especially DPPC, and were protein-free. Robillard et al. treated preterm infants with aerosolized DPPC in a mixture of propylene glycol and water (16) and Morley et al. tested endotracheal insufflation of ALEC, a dry powder surfactant composed of two phospholipids (DPPC:PG 7:3 by weight) (17), but both approaches were of limited success.

Colfosceril palmitate (EXOSURF^®^) consisted of DPPC with hexadecanol to promote adsorption and tyloxapol to facilitate dispersion (DPPC: hexadecanol: tyloxapol 85:9:6 by weight) and though this second-generation synthetic surfactant was efficacious in preterm infants with RDS, it was inferior to animal-derived surfactants in clinical trials and ultimately withdrawn from the market (18). Lucinactant (SURFAXIN^®^, Discovery Labs, King of Prussia, PA) combined KL4, a simplified peptide mimic of SP-B, and a phospholipid mixture closer to human composition (DPPC:POPG:Palmitic Acid:KL4 with 34 mg of lipids/mL and 0.86 mg of KL4/mL) (19). Although this surfactant performed well in clinical trials, it was pulled from the market because of stability and gel formation issues.

The various first- and second-generation synthetic surfactants did not compare well with animal-derived surfactants that rapidly improve oxygenation, reduce the need for ventilatory support, and decrease mortality in preterm infants with RDS (20). Addition of advanced SP-B and SP-C peptide mimics based on the amino acid sequence and structure of native proteins can correct these flaws (21), as shown for example in a rabbit RDS model (22). Chiesi Farmaceutici (Parma, Italy), the manufacturer of the porcine surfactant poractant alfa (CUROSURF^®^), developed CHF5633 (DPPC:POPG 1:1 with 0.2% Mini-B-leucine and 1.5% SP-C33) as the first third-generation synthetic surfactant with both a SP-B and a SP-C peptide mimic for clinical use. Mini-B is a 34-amino acid, disulfide-linked construct based on the primary sequences of the N- and C-terminal domains without the insertion sequence (23) and SP-C33 is a poly-leucine analog of SP-C (24). Despite its extremely low SP-B peptide mimic content, in vitro functional and structural characterization (25) and clinical tests were favorable and demonstrated safety and efficacy (26). After an impressive phase 2 trial (27), the compound was withdrawn from further testing because of its high production costs.

**Figure 1.**
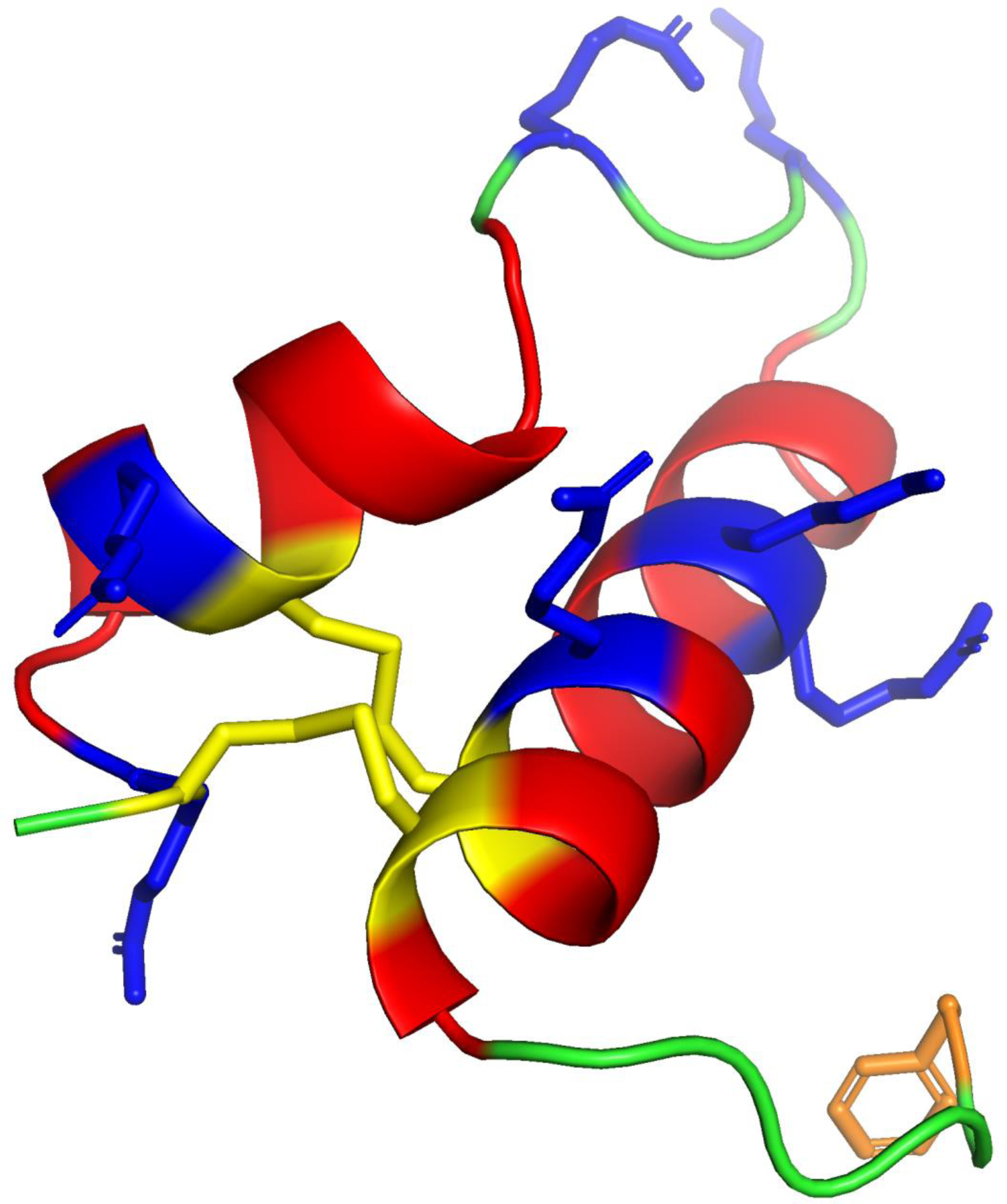
**Molecular illustration of the Super Mini B (SMB) peptide construct of the Saposin Surfactant Protein B (SP-B)** derived from atomic coordinates deposited in the ModelArchive (https://modelarchive.org), accession code: ma-abz44. SMB has an amphipathic helix hairpin structure that emulates critical conformational elements of the Saposin fold associated with the parent SP-B protein (13). The N-terminal and C-terminal alpha helical domains of the peptide are highlighted in red with polar charged amino acids lysine and arginine in blue. The bend domain is shown in green with charged polar residues in blue while N-terminal phenylalanine of the insertion sequence is highlighted in orange. In the disulfide linked SMB peptide, cystine residues are in yellow showing the N-terminal – C-terminal covalent linked connectivity (Cys 8 – Cys 40; Cys 11 – Cys 34).

**Figure 2.**
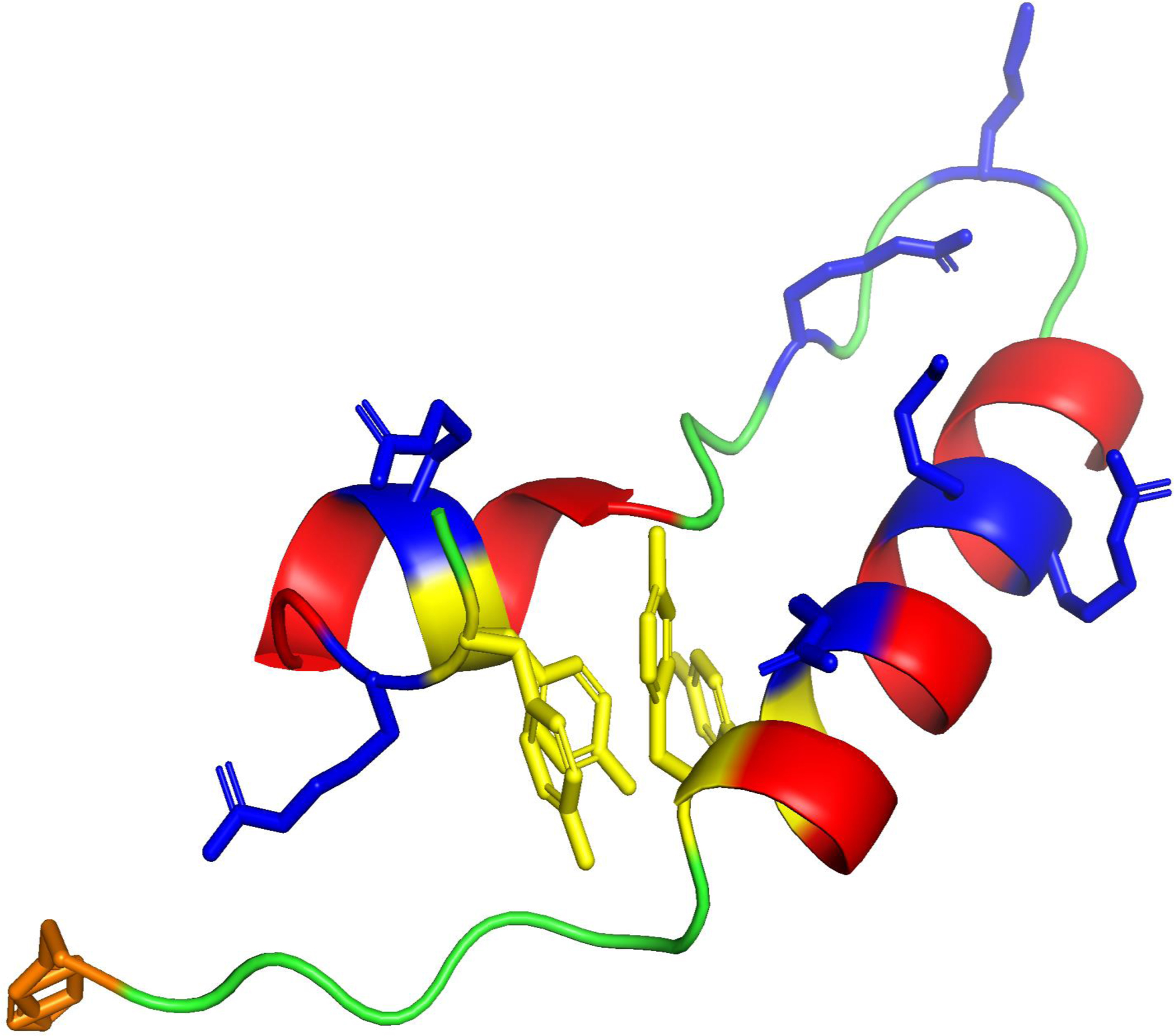
**Molecular illustration of the B-YL peptide construct of the Saposin Surfactant Protein B (SP-B)** derived from atomic coordinates deposited in the ModelArchive (https://modelarchive.org), accession code: ma-vilb7–2. B-YL has an amphipathic helix hairpin structure that emulates critical conformational elements of the Saposin fold associated with the parent SP-B protein (28). The N-terminal and C-terminal alpha helical domains of the peptide are highlighted in red with polar charged amino acids lysine and arginine in blue. The bend domain is shown in green with charged polar residues in blue while N-terminal phenylalanine of the insertion sequence is highlighted in orange. The sulfur-free peptide B-YL has the helix hairpin structure stabilized by replacing all cysteine residues with tyrosine amino acids. These aromatic residue substitutions, highlighted in yellow, facilitate the non-covalent hydrophobic interaction of adjacent tyrosine aromatic rings that stabilize the helix-hairpin structure, thereby mimicking the disulfide connectivity of the SMB construct.

Considering the crucial importance of SP-B in native surfactant, our research group has focused on the generation of third-generation synthetic surfactants based on human SP-B peptide mimics. SP-B is a small lipid-associating 79-amino acid monomer with a molecular weight of 8.7 kDa that belongs to the Saposin protein superfamily and is found in the mammalian lung as a covalently linked homodimer through a disulfide bridge. Each SP-B monomer contains three intramolecular disulfide bridges. Super Mini-B (SMB) is a 41-amino acid peptide construct based on the N- and C-domains of SP-B covalently joined with a turn and two disulfides that folds as an α-helix hairpin mimicking the properties of these domains in SP-B (Figure 1). It differs from its predecessor Mini-B as it has native SP-B residues 1–7, the SP-B insertion sequence, attached to the N-terminus of Mini-B (13). B-YL is a sulfur-free, non-covalent, aromatic ring side chain interaction stabilized SMB variant that has its four cysteine and two methionine residues replaced by tyrosine and leucine, respectively, (Figure 2) and does not need the oxidation step to produce a surface-active, α-helix hairpin (28). Liquid surfactant phospholipid formulations with 3% SMB (MINISURF^®^, Molecular Express, Rancho Dominguez, CA) (13) or B-YL (Synthsurf, Aerogen Pharma, San Mateo, CA) (28, 29) have been tested in ventilated, lavaged, surfactant-deficient rabbits and are as efficient in improving oxygenation and lung compliance as animal-derived surfactants.

**Figure 3.**
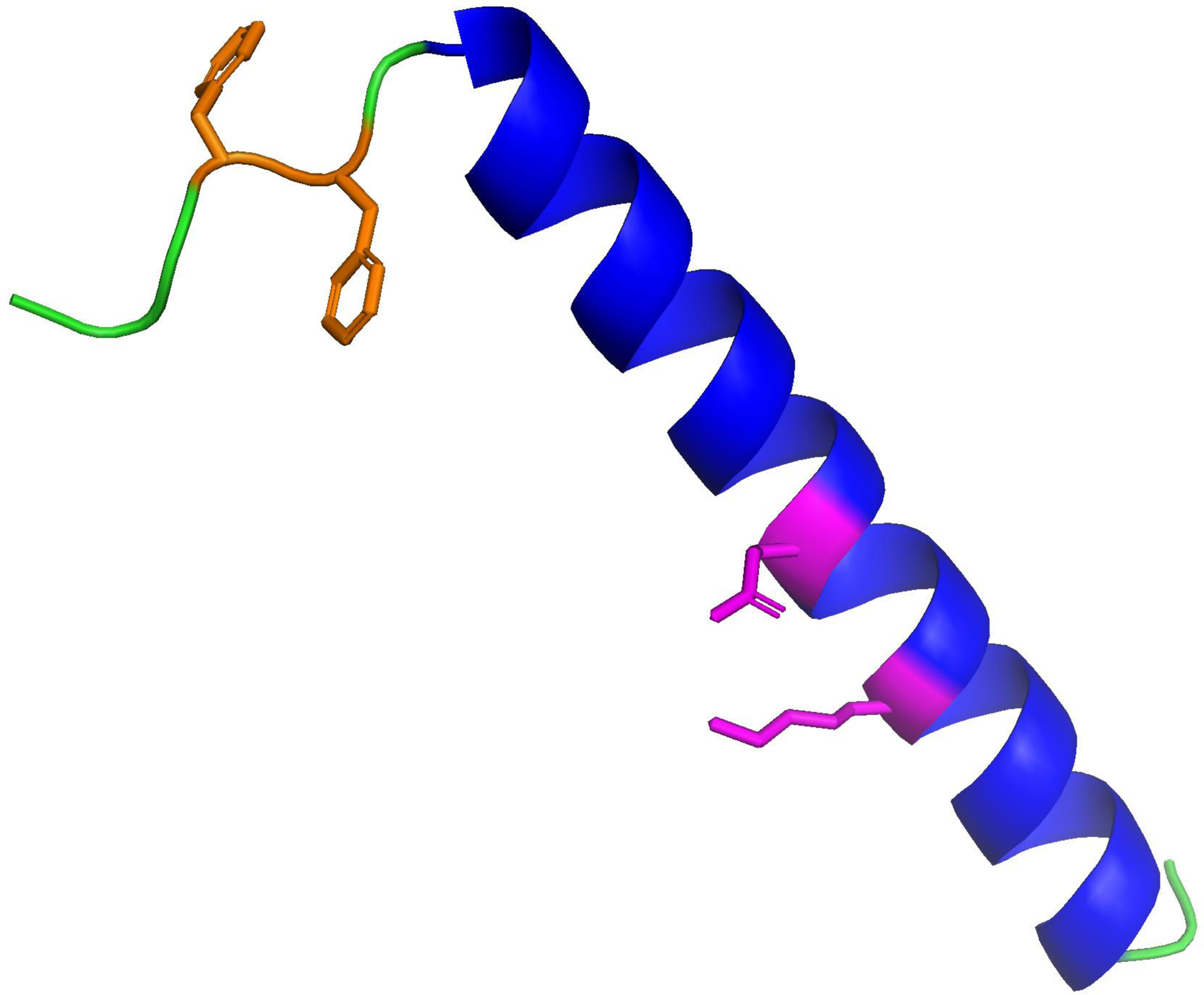
Molecular illustration of the secondary structure prediction for the Canine SP-C Ion-Lock Protein. Canine SP-C protein with phenylalanine substituted for cysteine-palmitic acid at amino acid position 4 in orange along with native sequence phenylalanine in position 5 and ion lock residue ion-lock pair E – K in magenta highlight at residues 20–24 in the hydrophobic poly-valine alpha helical amino acid sequence shown in blue. Bend and disordered domains are colored in green (35).

Although SP-C mutations are not lethal in the neonatal period, deficiency of this small hydrophobic protein (35-amino acid monomer with a molecular weight of s3.7 kDa) may give rise to childhood interstitial lung disease later in life (4). Synthetic surfactant with SP-B and SP-C peptide mimics is superior to single-peptide surfactants (22), but production of a stable SP-C peptide mimic has been hindered by its tendency to convert into beta-sheet aggregates, forming amyloid fibrils (30). Current clinical surfactant dispersions that are harvested from native sources have a limited shelf life. The temporal nature of these formulations may in large part be secondary to the formation of inactive amyloid SP-C protein. Usability duration of animal-derived surfactant formulations is limited to one year since the SP-C protein becomes deacylated due to loss of thioester linkages of the vicinal cysteine residues in the N-terminal that covalently link palmitoyl chains to the protein. Deacylation results in conversion of the hydrophobic alpha helix into antiparallel beta sheets that with time forms inactive amyloid fibers (31, 32). Synthetic SP-C proteins have been designed to overcome the effects of deacylation by substituting amino acid residues that stabilize the hydrophobic helix. One strategy for helix stabilization is the substitution of leucine residues for valine amnio acids that have a greater helical propensity to form stable helical conformations (25, 33). Another protein engineering approach to SP-C helical stabilization has been the substitution of polar residues that form ion-pairs in the hydrophobic domain. By strategically substituting these polar residue ion-pairs in the poly-valine helix, they form an ion-lock in the hydrophobic environment of the multilayer ensemble, thereby stabilizing the alpha helical conformation of the protein (Figure 3) (34, 35). The stable helical SP-C33-leucine design (24) has been further extended to include a SP-B mimic domain at the SP-C terminus, thereby creating a combination of the important elements of SP-B and SP-C proteins structure and function in a single combination surfactant protein (36).

The clinical switch to more frequent use of non-invasive nCPAP support for preterm infants with RDS has also increased research interest in aerosol delivery of surfactant and led to evaluation dry powder (DP) inhalation of synthetic surfactant in preclinical studies (37). We have tested aerosol delivery of various highly surface active spray-dried synthetic surfactant formulations (composed of KL4, SMB or B-YL peptide, mixed in 2 or 3 phospholipids [DPPC:POPG or DPPC:POPC:POPG], and lactose or trehalose and NaCl as excipients) in surfactant-deficient rabbits and preterm lambs, supported with mechanical ventilation or nCPAP, and shown the potential of this approach (14, 38). A combination of these novel DP synthetic surfactants and aerosol delivery systems with breath synchronization resulted in even more efficient lung dosing in surfactant-deficient animals (39). The surfactant group of Hindle and Longest at Virginia Commonwealth University has added to this development by adding mannitol and NaCl as hygroscopic excipients and l-leucine or trileucine as dispersion enhancer to the peptide-lipid mixture (40). This excipient enhanced growth (EEG) surfactant showed efficient aerosol delivery from a low dispersion air volume dry powder inhaler and may boost lung dosing efficacy.

Jardine et al. (41) recently demonstrated that administration of aerosolized SF-RI 1 surfactant to preterm infants with RDS via nCPAP and a prototype breath synchronization device (AEROFACT^®^, Aerogen Pharma, San Mateo, CA) is safe and holds considerable promise for the future of non-invasive newborn respiratory care. The planned multicenter neonatal interventional randomized controlled trial of nebulized surfactant for preterm infants with respiratory distress (Neo-INSPIRe) trial (42) will expand clinical experience with this aerosolized surfactant/device combination.

## OFF-LABEL SURFACTANT USE

Although clinical, animal-derived surfactant is only approved by the FDA for treatment of preterm infants with RDS due to lung immaturity, there is good evidence for “off-label” surfactant treatment in term infants with respiratory failure due to meconium aspiration syndrome or sepsis/pneumonia, i.e. parenchymal lung disease leading to surfactant insufficiency due to surfactant inactivation (43, 44). Surfactant treatment in children and adults with acute respiratory disease syndrome (ARDS) has so far met with limited success (45). Recent in vitro studies on synthetic surfactant use in Covid-19 pneumonia showed strong binding of the SP-B peptide mimic SMB to the angiotensin-converting enzyme 2 (ACE-2) receptor that Covid-19 uses as port of cellular entry (46). This observation corresponds with the findings in a small study of Covid-19 positive ARDS patients that surfactant instillation reduced 28-day mortality (47) and suggests a potential role for surfactant therapy in viral pneumonia.

## SYNTHETIC SURFACTANT AS A DRUG-DELIVERY SYSTEM

Synthetic surfactants have gathered substantial attention as a potential drug-delivery system due to their amphiphilic properties that allow them to solubilize hydrophobic drugs, enhance their bioavailability, and carry combination therapies. As synthetic surfactant is versatile and tunable, modifications in physicochemical properties can enable target delivery, provide controlled release, and meet specific drug delivery requirements.

Systemic postnatal corticosteroid therapy reduces the risk of developing bronchopulmonary dysplasia in preterm infants with ventilatory support for RDS (48), but also leads to an increased incidence cerebral palsy if used in the first week after birth. The risk of cerebral palsy with early dexamethasone treatment can be circumvented by intrapulmonary instead of systemic therapy and has raised interest in the addition of corticosteroids, especially budesonide, to surfactant formulations (49) used for intratracheal treatment of RDS in preterm infants (25, 50). Hidalgo et al. (51) studied adsorption and surface activity of corticosteroids mixed in poractant alfa and the synthetic surfactant CHF5633 and found no interference, thereby confirming the potential of this approach. The randomized PLUSS “Preventing Lung Disease Using Surfactant + Steroid” trial in 18 neonatal centers from Australia, New Zealand, Singapore, and Canada, designed to better define the outcome of the combination of surfactant and steroids (52), has just completed recruitment after enrolling 1,062 babies. The results of the PLUSS are pending and will have a high impact on the clinical application of this combination therapy.

Another drug of interest for intrapulmonary delivery with synthetic lung surfactant is the nuclear transcription factor Peroxisome Proliferator-Activated Receptor gamma (PPARƴ). PPARƴ plays a pivotal role in physiologic lung development and homeostasis by stimulating alveolar interstitial lipofibroblast maturation (53). Nebulization of a mixture of the PPARƴ-agonist pioglitazone and synthetic B-YL surfactant (3% of B-YL in DPPC:POPC:POPG 5:3:2 by weight) accelerated lung maturation in newborn rats and prevented neonatal hyperoxia-induced lung injury more than either modality alone, demonstrating the potential of this combination to provide more effective prevention of bronchopulmonary dysplasia (54).

## CONCLUSION

Synthetic surfactant therapy is revolutionizing the treatment of RDS and will significantly improve survival rates for premature infants. Currently available third-generation synthetic lung surfactant formulations based on peptide mimics of SP-B and C are as surface active as animal-derived clinical surfactants and clearly have a definite potential to replace the latter in clinical practice soon. CHF5633 (with SP-B and SP-C mimics) was very effective in studies in preterm infants with RDS, but withdrawn because of its high production costs. Production costs of MINISURF^®^ and Synthsurf, using only SP-B peptide mimics in a phospholipid mixture, are much lower and projected at <$50 per 100 mg dose (instead of ~$500 for the same amount of an animal-derived surfactant) and the required preclinical studies for drug approval are advancing steadily. Addition of advanced SP-C peptide mimics with a preserved alpha-helical structure or use of SP-B and C combinations may also assist with stabilization of surfactant function.

Aerosol delivery during nCPAP support has also made a lot of progress in clinical practice and the combination of advanced nebulizers and synthetic surfactant works well in ongoing (pre)clinical studies. Translation of these findings to the nurseries of low- and middle-income countries is a major goal of the Gates Foundation as lack of nCPAP and surfactant are drivers of neonatal mortality (55).

## FUTURE DEVELOPMENT

Ongoing research aims to refine existing therapies and explore novel delivery approaches. Key areas for future development of synthetic lung surfactant are personalized medicine, genetically engineered surfactants, combination therapies, and improved delivery methods.

Optimal treatment of neonatal RDS is complex and requires personalized medicine based on pathophysiology and actual patient needs. This involves techniques such as surfactant biological tests (e.g., adsorption and stable microbubble testing), lung mechanics (e.g., compliance and work of breathing), lung aeration (e.g., lung ultrasound and electrical impedance tomography), and gas exchange metrics (e.g., transcutaneous partial pressure of oxygen measurements) (56, 57).

Recombinant production of SP-B and SP-C is complicated by the hydrophobicity of these transmembrane proteins, including combinations of SP-B and SP-C (33, 36). Gene-editing for correction of SP-B deficiency may in the near future replace and avoid lung transplantation, currently the only treatment option for SP-B mutations (58).

Aerosol surfactant delivery (37) and laryngeal mask airway surfactant administration (11) are receiving increasing research interest, but practical introduction of these techniques in intensive care of preterm infants needs more research.

## Figures and Tables

**Table 1:** Advantages of synthetic over animal-derived lung surfactant.

	Synthetic surfactant	Animal-derived surfactant
**Purity**	Well-defined chemicals without contaminants or pathogens	Produced by washing or extracting animal lungs
**Consistency**	Uniform formulation, consistent quality, and performance	Inter-batch variation in composition and physical properties
**Safety**	No risk of infectious diseases	May transmit zoonotic diseases
**Allergic reactions or immune responses**	No risk of allergic reactions	Potential risk due to animal-derived proteins
**Customization**	Can be adapted to improve efficacy and safety	Cannot be easily changed
**Shelf-life**	>3 years	1 year
**Production and availability**	Efficient production at a large scale	Production depends on availability of suitable livestock
**Pricing**	Depends on composition, but relatively cheap	Expensive due to extensive quality control measures
**Acceptance**	Solution when use of animal-derived surfactant is unacceptable	May be unacceptable due to ethical, religious, cultural, and/or environmental concerns

**Table 2:** Abbreviations

ARDS	Acute respiratory distress syndrome
B-YL	Modified SMB peptide
CHF5633	Chiesi Farmaceutici synthetic surfactant
Covid-19	Coronavirus disease 2019
DP	Dry powder
DPPC	1,2-dipalmitoyl-sn-glycero-3-phosphocholine
EEG surfactant	Excipient enhanced growth surfactant
INSURE	Intubation-surfactant-extubation
KL4	Sinapultide peptide (SP-B mimic)
LISA	Less invasive surfactant administration
MIST	Minimally invasive surfactant therapy
nCPAP	nasal continuous positive airway pressure
PG	Phosphatidylglycerol
PLUSS	Preventing lung disease using surfactant + steroid
POPC	1-palmitoyl-2-oleoyl-sn-glycero-3-phosphocholine
POPG	1-palmitoyl-2-oleoyl-sn-glycero-3-phosphoglycerol
PPAR-γ	Peroxisome proliferator-activated receptor gamma
RDS	Respiratory distress syndrome
SF-RI 1	Bovine surfactant
SMB	Super Mini-B peptide (SP-B mimic)
SP	Surfactant protein
